# A Robust Fire Detection Model via Convolution Neural Networks for Intelligent Robot Vision Sensing

**DOI:** 10.3390/s22082929

**Published:** 2022-04-11

**Authors:** Qing An, Xijiang Chen, Junqian Zhang, Ruizhe Shi, Yuanjun Yang, Wei Huang

**Affiliations:** 1 School of Artificial Intelligence, Wuchang University of Technology, Wuhan 430223, China; 120160450@wut.edu.cn (Q.A.); 120150187@wut.edu.cn (Y.Y.); 120100398@wut.edu.cn (W.H.); 2 School of Safety Science and Emergency Management, Wuhan University of Technology, Wuhan 430079, China; zhangjunqian@whut.edu.cn (J.Z.); srz3105187674@whut.edu.cn (R.S.)

**Keywords:** YOLOv5, deep learning, detection, dynamic convolution

## Abstract

Accurate fire identification can help to control fires. Traditional fire detection methods are mainly based on temperature or smoke detectors. These detectors are susceptible to damage or interference from the outside environment. Meanwhile, most of the current deep learning methods are less discriminative with respect to dynamic fire and have lower detection precision when a fire changes. Therefore, we propose a dynamic convolution YOLOv5 fire detection method using a video sequence. Our method first uses the K-mean++ algorithm to optimize anchor box clustering; this significantly reduces the rate of classification error. Then, the dynamic convolution is introduced into the convolution layer of YOLOv5. Finally, pruning of the network heads of YOLOv5’s neck and head is carried out to improve the detection speed. Experimental results verify that the proposed dynamic convolution YOLOv5 fire detection method demonstrates better performance than the YOLOv5 method in recall, precision and F1-score. In particular, compared with three other deep learning methods, the precision of the proposed algorithm is improved by 13.7%, 10.8% and 6.1%, respectively, while the F1-score is improved by 15.8%, 12% and 3.8%, respectively. The method described in this paper is applicable not only to short-range indoor fire identification but also to long-range outdoor fire detection.

## 1. Introduction

Currently, temperature or smoke detectors are mainly used to detect fire. One disadvantage of these sensor-based fire detection methods is that they are expensive. Furthermore, these sensor-based methods can only detect indoor fires. Cameras, on the other hand, are found everywhere in China, and they have been used to detect objects [1] and track targets [2,3]. Several methods have been proposed to detect fires or smoke using traditional video-surveillance cameras.

Image-based fire detection methods mainly include the traditional color-based and deep learning methods. Color-based methods need to consider the color of the flames, and different types of fires produce different flame colors. The popular color-based object detection methods mainly include color spaces, HSV [4], L*a*b [5], YUV [6] and YCbCr [7]. Meanwhile, the conventional fire detection algorithm uses flame features to conduct fire identification. However, it is difficult to define flame features. Recently, convolutional neural networks (CNNs) [1] have been widely used in object detection. In view of this, Luo et al. [8] used a CNN for video fire and smoke detection. In the early stages of fire detection, the researchers trained fire images by using a constructed CNN based on scale constraints [9]. However, the CNN requires a huge amount of data to train and takes a lot of time [10,11]. In view of this, transfer learning was utilized to design neural networks, and the AlexNet-based CNN [12] and YOLO-based CNN [13] have been proposed to conduct fire detection.

At present, the task of object detection mainly functions by selecting the appropriate detection algorithm according to different application scenarios. Object recognition methods include one-stage and multi-stage algorithms. However, for existing deep learning object recognition methods, it is difficult to obtain high precision and speed in a small model. Considering the dynamic characteristics of fire, we propose a dynamic convolution YOLOv5 algorithm. First, we propose using the K-mean++ algorithm to optimize anchor box clustering so as to be suitable for a small target dataset and improve detection accuracy. Second, dynamic convolution is introduced into the convolution layer of YOLOv5 to conduct the adjustment of convolution parameters to further improve detection accuracy. Finally, the neck and head layers in YOLOv5 are pruned to remove the large target detection layer. Consequently, the inference speed is improved while maintaining high detection accuracy.

The main contents of this paper include:

(1) The K-mean++ algorithm is proposed to optimize anchor box clustering and significantly reduce the rate of error of the classification results.
(2) Dynamic convolution is introduced in the convolution layer of YOLOv5 based on the determination of the candidate region box by K-means++.
(3) The network heads of YOLOv5’s neck and head are pruned to improve detection speed and further achieve the objective of real-time detection while ensuring accuracy.

The remainder of this paper is organized as follows: Section 2 introduces related work on fire detection based on image color and CNN methods. Section 3 constructs our dynamic convolution YOLOv5 fire detection method, and Section 4 shows the performance comparison between our proposed method and other methods. Finally, Section 5 summarizes the paper. 

## 2. Related Work

There are various visual methods that have been proposed to detect fire; these mainly include image color methods and CNN methods. Most of the image color methods use a combination of different colors to conduct fire detection because this is a simple way to identify fire pixels. Recently, in addition to successful applications of deep learning (DL) methods in the fields of natural language processing [14,15] and image classification [16,17], there has also been considerable progress achieved in DL-based fire detection methods [18]. 

### 2.1. Conventional Image-Based Methods

Smoke is the dominant feature of early-stage fires. Some researchers focus on smoke detection in the early stages of a fire. Calderara et al. [19] used the energy function to extract smoke color information and achieve fire detection. Their method was suitable not only for fire detection in the daytime but also at night; it used a camera to detect fires in an area 80 m^2^. Yuan used smoke contours and spectral features to conduct fire detection [20]; this method has better generalization performance and less insensitivity to geometry transform. Considering that smoke color has the characteristics of semi-transparency and diffusion, smoke can be detected by the spatio-temporal variation trends of color [21]. However, color-based smoke detection is not entirely reliable because some smoke colors, such as gray and black, are common in daily life. To improve smoke detection performance, some researchers combined color, texture and dynamic features to implement smoke identification [22,23]. Ye et al. [24] used a pyramid model to conduct the multi-scale decomposition of fire images and combined this with the use of an SVM to implement smoke detection. The advantage of this method is that it considers the spatial and temporal information of image sequences simultaneously, so the accuracy of fire detection can be improved. In addition, fire and smoke have common motion characteristics. Therefore, Shiping et al. [25] took advantage of this characteristic to detect smoke. Simultaneously, Islam et al. used the characteristics of smoke color and dynamics to detect smoke [26]. Their method achieved 97.3% fire classification accuracy; however, it cannot be applied for accidental fires far away from cameras. Since the fire combustion process involves color, shape changing and motion, another real-time fire detection method was constructed by using a combination of these features [27]. Premal et al. [28] separated flame and high-temperature fire center pixels by using the statistical parameters of fire, color and space. Static and dynamic texture characteristics are the main characteristics in the process of fire combustion. Candidate flame regions can be identified by the YCbCr color space. From candidate flame regions, forest fires can be detected according to the static and dynamic texture features [29]. Similarly, Han et al. combined Gaussian-mixture-model-based background subtraction and multi-color features to obtain the motion features and color information of fire [30]. Previous fire detection methods based on color information need to conduct tuning of many parameters, which influences the stability of fire detection. In order to reduce the impact of parameter tuning, a novel fire detection approach based on Red Green Blue and CIE L*a*b color models was proposed [31].

### 2.2. Deep-Learning-Based Methods

The conventional image-based methods are easily influenced by fire-like objects and are prone to false alarms. To address these false alarms, CNNs have been applied to fire detection. For example, a DCNN architecture was designed to detect forest fires [32]; the model demonstrated anti-noise performance in the field of fire detection. A CNN needs to be trained before it is used for fire detection. Some researchers used many fine-grained images to train the CNN model and verify that it could be used for fire detection [33]. CNNs demonstrate good performance in object classification, feature extraction and target recognition. However, they still result in some false positives. To reduce false alarms in object recognition, the Region of Interest (ROI) was proposed for the process of fire detection [34]. CNN methods generally cost more computational time and memory when applied to object identification. Muhammad et al. [35] applied GoogLeNet to construct a cost-effective deep learning architecture for fire detection. GoogLeNet is significantly more efficient than VGGNet, with only about five million parameters. In order to improve dynamic fire detection ability, Chen et al. [36] first segmented the flame features and then used combining random gradient descent and momentum correction to train the CNN. Muhammad et al. [37] used adaptive optimization mechanisms to obtain a fine-tuned CNN for early fire detection. Dunnings et al. [38] improved the GoogLeNet model for detecting fire; their network architecture was of significantly reduced complexity. Jadon et al. [39] designed a lightweight, 14-layer CNN to conduct fire and smoke detection. Saeed et al. [40] first used Adaboost-MLP to forecast fires and then proposed the Adaboost-LBP model and a CNN to detect fires. Muhammad et al. [41] used a lightweight CNN to detect fires; it balances accuracy and running time. Zhang et al. [42] utilized a contextual-based CNN for forest fire detection, and the hyperparameters of the fire detection mode were optimized. Thus, the accuracy of fire detection has improved. Recently, Ross et al. [43] proposed Faster R-CNN: this algorithm uses Region Proposal Networks (RPN) to replace the selective search algorithm, thus reducing calculation time while ensuring accuracy. Faster R-CNN mainly includes a convolution layer, a candidate box recommendation network, a feature aggregation layer and a classification layer. The model can realize feature extraction, candidate box extraction, border regression and classification integration. However, it needs to classify each candidate box, so it needs to carry out a lot of operations. Meanwhile, it also has the problem of the registration of the original image and the feature image, which affects the accuracy of the model. Li et al. [44] compared the performance of Faster-RCNN, R-FCN, SSD and YOLOv3 in fire detection and analyzed the advantages and disadvantages of each of these methods. Results show that the performance of YOLOv3 is superior to the other three methods. The average precision of fire detection is higher than the other methods, and it has stronger robustness of detection. To detect fires using deep learning, it is necessary to balance accuracy, model size and speed. In view of this, Li et al. [45] used the fusion mechanism to balance accuracy, model size and speed. In order to improve the fire detection efficiency of a convolutional neural network, the convolutional kernel parameters and dense layers were calculated, and those fires with a low energy impulse response were eliminated [46]. Another type of convolutional neural network is YOLO (You Only Look Once) [47], and it consists of four components: an input terminal, a backbone network, a neck network and a detection header. This model strengthens the network feature fusion and reduces the problem of small target feature loss. This type of model saves a lot of training time and improves detection speed. 

## 3. Proposed Method

### 3.1. Determination of Anchor Box Based on K-Means++

The YOLOv5 model introduces the use of an anchor box in the object detection process. An anchor box is a group of initial regions with a fixed size and aspect ratio. In the process of training, the closer the initial anchor box is to the real boundary box, the more easily the model will be trained, and the more consistent with the real boundary box the predicted boundary box will be. Therefore, the anchor parameters in the original YOLOv5 model need to be adjusted according to the training requirements of different datasets. According to the characteristics of the YOLOv5 model, the width and height of 9 clustering centers need to be found; they are then used as the values for the anchor parameters in the network configuration file. Due to the simple and efficient characteristics of K-means, it has been used in the field of clustering. The YOLOv5 model uses the K-means clustering algorithm to obtain k initial anchor boxes. However, the disadvantage of the K-means algorithm is that the number of clustering centers, k, and the initial clustering center need to be given in advance. Yet, it is difficult to determine the clustering centers and the initial clustering center in advance. To address the defects of K-means algorithm, the K-means++ algorithm is used in this paper to obtain k initial anchor boxes. K-means++ optimizes the selection of the initial point and can, therefore, significantly reduce the rate of classification error so as to obtain an anchor box size more suitable for the detection of small objects. 

The specific process of determining an anchor box based on K-means++ is as shown below:

(1) A sample is randomly selected as the first clustering center.
(2) The distance between each sample and the nearest cluster center is calculated, and Equation (1) is used to calculate the probability of each sample being selected as the next cluster center:
  (1) $$\frac{D{\left(x\right)}^{2}}{\sum_{x\in X}D{\left(x\right)}^{2}}$$

The next cluster center is selected by the roulette wheel method. Step (2) is repeated until k clustering centers are selected.

### 3.2. Dynamic Convolution YOLOv5

The performance of lightweight convolutional neural networks (CNNs) decreases because their low computational budgets constrain both the depth and the width of CNNs, resulting in a limited representation ability. To solve this problem, Chen, et al. [48] proposed dynamic convolution, which increases model complexity without increasing the network depth or width. 

The traditional static perceptron is described by an activation function, which can be written as:

(2) $$y=g\left(W^{T}x+b\right)$$

where W is weight matrix and b is bias vector.

The dynamic perceptron is defined by aggregating multiple linear functions and can be described as:

(3) $$\left\{\begin{array}{c} y=g\left({\tilde{W}}^{T}\left(x\right)x+\tilde{b}\left(x\right)\right)\\ \tilde{W}\left(x\right)=\sum_{k=1}^{K}\pi_{k}\left(x\right){\tilde{W}}_{k},\;\tilde{b}\left(x\right)=\sum_{k=1}^{K}\pi_{k}\left(x\right){\tilde{b}}_{k},\\ s.t.\;0\leq \pi_{k}\left(x\right)\leq 1,\sum_{k=1}^{K}\pi_{k}\left(x\right)=1\; \end{array}\right.$$

where $\pi_{k}$ is the attention weight for the kth linear function $\tilde{W_{k}^{T}}x+{\tilde{b}}_{k}$.

**Figure 1:**
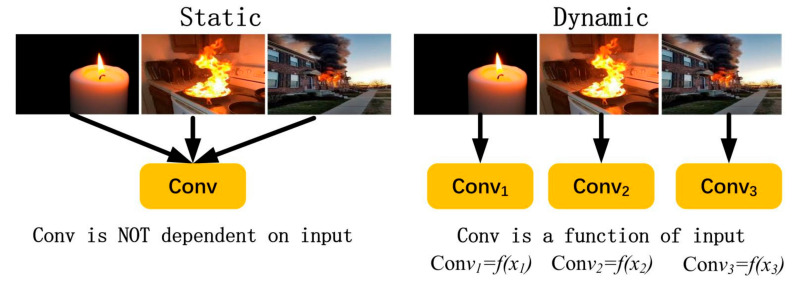
Static and dynamic convolution.

According to the dynamic perceptron model, dynamic convolution has k convolution kernels. They are aggregated by using the attention weights {π_k_}. To build a dynamic convolution layer, batch normalization and an activation function are adopted after the aggregated convolution. The basic idea of dynamic convolution is to adjust the kernel parameters adaptively according to the different input images. Static convolution uses the same convolution to perform the same operation for all input images, while dynamic convolution adjusts different images and uses more appropriate convolution parameters for different input images, as shown in Figure 1.

Dynamic convolution is a dynamic aggregation method based on multiple parallel convolution kernels. Attention dynamically adjusts the weight of each convolution kernel according to the input so as to generate adaptive dynamic convolution. The representation power is improved by assembling multiple kernels because these kernels are aggregated in a non-linear way via attention. Dynamic networks introduce two additional computations: the superposition of the attention model and the convolution kernel. The attention model is composed of an AVG pool and two-layer full convolution with low computational complexity. The small amount of extra computation combined with the significant expressional power makes dynamic convolution very suitable for lightweight neural networks. 

**Figure 2:**
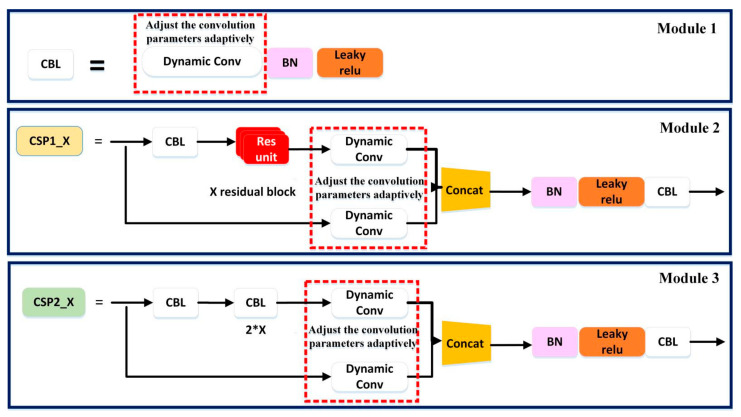
Improved YOLOv5 convolution layers with dynamic convolution.

In this paper, we introduce dynamic convolution into the YOLOv5 fire detection model. All of the ordinary convolution layers in the YOLOv5 network structure are replaced with dynamic convolution layers. The structure of this model is mainly composed of module 1, module 2 and module 3, as shown in Figure 2.

(1) Module 1 is the CBL module, which is the smallest component in the YOLOv5 network structure. The CBL module consists of the Conv + BN + Leaky_relu activation functions, which are replaced by the Dynamic Conv + BN + Leaky_relu activation functions, as shown in Module 1 of Figure 2. The role of the CBL module is that it uses the activation function for convolution.
(2) Module 2 is the CSP1_X module. It is used in the backbone network, which can increase the residual structure, thus increasing the gradient value of backpropagation between layers. Thus, the loss of gradient due to deepening is avoided, and finer-grained features can be extracted. The CSP1_X module consists of a CBL module, a RES unint module, a Conv and a Concat module, which are replaced by a CBLmodule, a Res unint module and a dynamic Conv and Concat, as shown in Module 2 of Figure 2.
(3) Module 3 is the CSP2_X module. It is located in the neck layer of the network structure. It consists of Conv and X Res unint modules and Concat, which are replaced by dynamic Conv and X Res unint modules and Concat, as shown in Module 3 of Figure 2.

### 3.3. Optimization of Network Structure Based on Structure Pruning

Pruning algorithms generally include unstructured and structured pruning. Unstructured pruning includes fine-grained pruning, vector-level pruning and kernel-level pruning; it achieves a certain balance between the number of parameters and the model performance. The disadvantage of this type of pruning algorithm is that when the topology of the network changes, a special algorithm needs to be designed to support this sparse algorithm. Structured pruning is mainly filter-level pruning, and the pruning algorithm only needs to change the filter banks and the number of feature channels in the network. The advantage of structured pruning is that it does not require the design of a special algorithm to perform its function, and it can achieve the pruning of the entire network layer. Therefore, structured pruning is used to prune the structure of the dynamic convolution YOLOv5 network in this paper.

**Figure 3:**
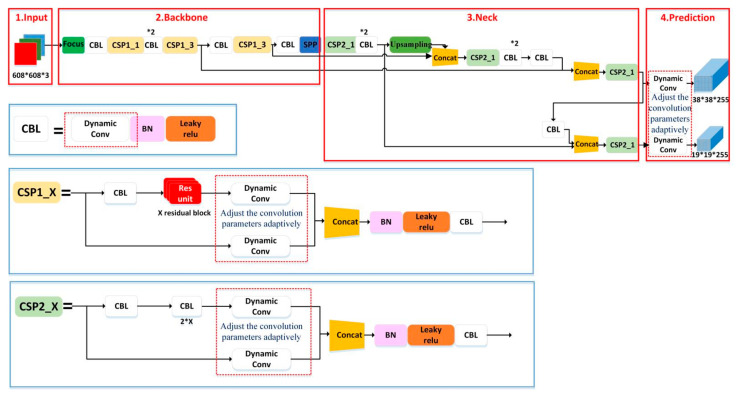
Dynamic convolution YOLOv5 network model structure.

In the neck part of the YOLOv5 network structure, three different cascade structures are used to form three detection heads. The output feature scales of the three detection heads are 76 × 76, 38 × 38 and 19 × 19, respectively; they are used to detect large, medium and small objects, respectively, in images. For fire detection, the accuracy and speed of small fire detection both need to be improved. However, the 76 × 76 detection head requires a lot of time, and it is not suitable for the improvement of inference speed. In this paper, structural pruning is proposed to prune the YOLOv5 neck part of the network, removing the large object detection heads, and reserving only the medium and small object detection heads, as shown in Figure 3.

## 4. Results and Discussion

### 4.1. Experimental Setup

(1) Experiment details: The experimental hardware is a server equipped with an Intel (R) Celeron (R) CPU N2840 @ 2.16 GHz, 4.00 GB RAM, and a 1080Ti graphics card that has 4 GB on-chip memory. The improved YOLOV5 network model is trained on the Pytorch deep learning framework.
(2) Datasets: the experimental dataset consists of three parts, namely a training, a verification and a test dataset, as shown in Table 1.

**Figure 4:**
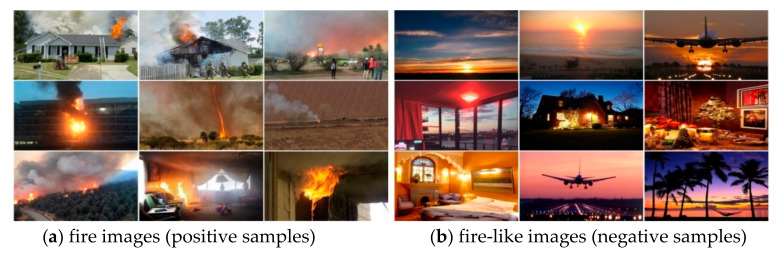
Training dataset.

The dataset is divided into fire images and non-fire images. The fire images are referred to as the positive sample, and the non-fire images are referred to as the negative sample. One part of the positive sample data in the training dataset is from the public flame datasets ImageNet and Bow-FIRE, and the other is from the internet. The fire region of each positive sample in the whole training dataset is manually labeled using the public labeling system. All sample images are processed and stored in the format of the Pasal Voc2007 sample set. The negative samples in the training dataset are all crawled from the internet, as shown in Figure 4. The positive samples in the test set are taken from videos in the Bilkent University Fire Database, and 2442 images are extracted from the videos. The negative samples in the test set are all crawled from the internet.

### 4.2. Evaluation Metrics

For fire detection, there are four possible detection results. If the image is fire and it is detected as fire, then the detection result is true positive (TP); if it is detected as non-fire, the detection result is false negative (FN). If the image is non-fire and it is detected as non-fire, the detection result is true negative (TN); if it is detected as fire, then the detection result is false positive (FP). All four possible fire detection outcomes are listed in Table 2.

The common evaluation metrics for object detection include precision, recall, accuracy and F1-score.

(4) $$P=\frac{\mathrm{TP}}{\mathrm{TP}+\mathrm{FP}}$$

(5) $$R=\frac{\mathrm{TP}}{\mathrm{TP}+\mathrm{FN}}$$

(6) $$\mathrm{Acc}=\frac{\mathrm{TP}+\mathrm{TN}}{\mathrm{TP}+\mathrm{FP}+\mathrm{TN}+\mathrm{FN}}$$

F1-score is the metric that is used to characterize the balance degree between recall and precision, which can be computed as:

(7) $$F1\text{-}\mathrm{score}=\frac{P*R}{P+R}$$

where P is the precision and R is recall.

### 4.3. Ablation Experiments

In order to verify that the dynamic convolution and structural pruning proposed in this paper have effects on the accuracy and speed of the YOLOv5 model, two ablation experiments are designed to verify their effectiveness.

#### 4.3.1. Dynamic Convolution Ablation Experiment 

**Figure 5:**
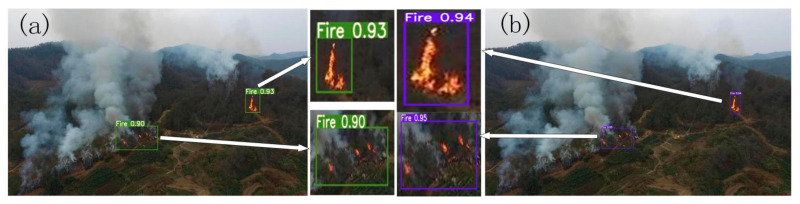
Fire detection results of YOLOv5 and the dynamic convolution YOLOv5. (**a**) YOLOv5. (**b**) Dynamic convolution YOLOv5.

We replace all static convolutions with dynamic convolutions in the three modules and construct the dynamic convolution YOLOv5 model. In order to verify the detection effect of dynamic convolution for multiple targets and remote small targets, we select multi-target and long-range fire images. We use these two models to implement the fire detection experiment and obtain the fire detection results, as shown in Figure 5. 

In Figure 5, it is clearly visible that the detection accuracies of YOLOv5 are 93% and 90%, while the detection accuracies of dynamic convolution YOLOv5 are 94% and 95%. Dynamic convolution has high accuracy and stability; the accuracy difference between the two object boxes is less than 1%, and both are higher than YOLOv5. It illustrates that dynamic convolution has the ability to adjust parameters adaptively, enabling it to detect small and long-distance targets with high accuracy. 

The four possible outcomes of fire identification, as listed in Table 2, are obtained. Then, we calculate the precision, recall, accuracy and F1-score according to Equations (4)–(7), as shown in Table 3. 

From Table 3, it is clearly visible that the precision, recall, accuracy and F1-score of dynamic convolution YOLOv5 are slightly greater than YOLOv5. After static convolution is replaced by dynamic convolution in all three modules, the accuracy of fire detection is improved. Although the detection speed of dynamic convolution YOLOv5 is lower, the detection time for each image is only 3 ms longer than YOLOv5. The detection precision of dynamic convolution YOLOv5 can be as high as 96.4%, which is 6.3% higher than that of YOLOv5 model; detection speed is sacrificed, but precision is greatly improved. 

#### 4.3.2. Pruning Experiment

In the dynamic convolution ablation experiment, the precision of fire detection improves, but the detection time also increases. In view of this, the neck portion of the YOLOv5 network is pruned. The large object detection head is removed, and only the medium and small object detection heads are retained. Consequently, the computation and model complexity can be greatly reduced, and the detection speed can be improved. We use YOLOv5 after pruning to conduct fire detection and compare it with YOLOv5 before pruning, as shown in Table 4.

Table 4 shows that the precision, recall, accuracy and F1-score of YOLOv5 after pruning are slightly lower than YOLOv5 before pruning. It illustrates that pruning definitely influences the effectiveness of fire detection. However, pruning has little effect on fire detection accuracy, according to the comparative analysis in Table 4. After pruning, the model size of YOLOv5 is reduced from 13.7 to 10.8. Importantly, the detection time for each image is reduced greatly, from 26 to 13 ms. Therefore, pruning can greatly reduce detection time with negligible precision loss. 

### 4.4. Performance Comparison

#### 4.4.1. Comparison of Training Results

In this experiment, the size of the input images is set as 640 × 640; the initial learning rate, moment and weight_decay are set as 0.001, 0.9 and 0.0005, respectively. The batch size and training epochs are set to 32 and 230, respectively. We use the K-means++ clustering algorithm to conduct the clustering of the anchor box and obtain six object boxes for two types of anchor box, described as:

(8) $$\mathrm{Anchor}\;\mathrm{Box}=\left\{\begin{array}{c} \left[10,14;23,27;37,58\right]\\ \left[81,82;135,169;344,319\right] \end{array}\right.$$

**Figure 6:**
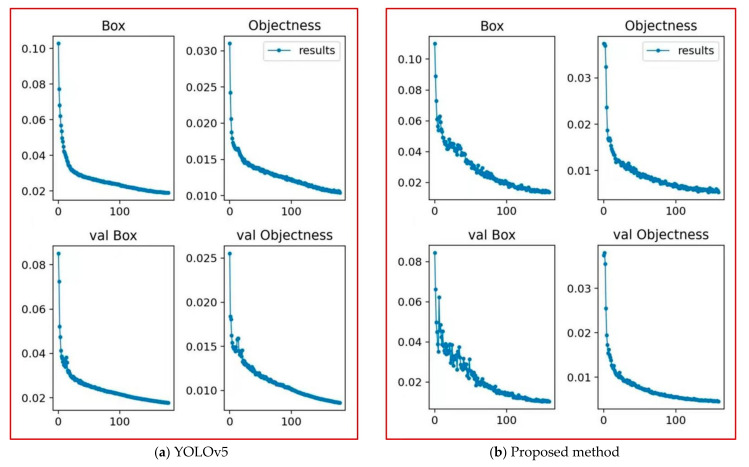
Comparison of the change curve between the loss value of the object box and the belief of the object category.

These two anchor boxes are used for the dynamic convolution YOLOv5 detection heads with different scales of 38 × 38 and 19 × 19, respectively, and for predicting the object bounding box. The training dataset is used to train both the YOLOv5 and our proposed method under the condition of 200 epochs, and the training results are shown in Figure 6.

**Figure 7:**
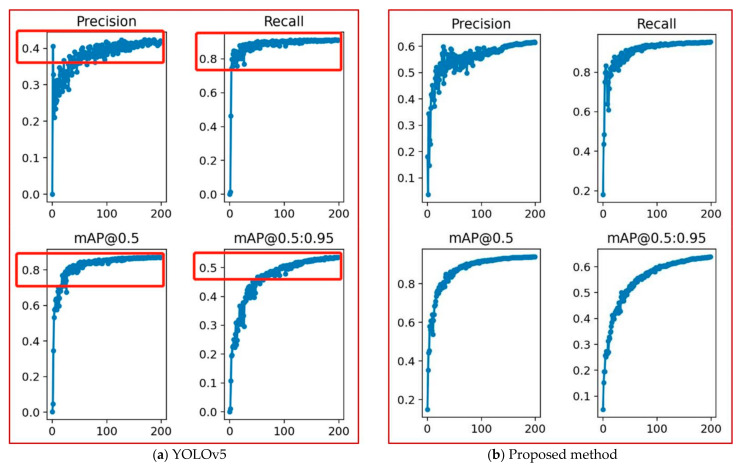
Changes in recall, precision and mAP of the YOLOv5 and the proposed method.

After training, the validation set is used to validate the model after epochs. The IOU threshold on the validation dataset is set to 0.5. Figure 7 shows the recall, precision and mAP obtained by the YOLOv5 and by our proposed method. It can be seen that the precision of the proposed method is 20% higher than that of the YOLOv5. The recall of the proposed method improved from 0.81 to 0.88 and increased by 7% compared with YOLOv5. mAp@0.5 improved from 0.81 to 0.89 and increased by 8%. mAp@0.5:0.95 improved from 0.52 to 0.62 and increased by 10%.

#### 4.4.2. Comparison in Different Scenarios Based on the Visualization

In order to reflect the reliability of the proposed method in different fire detection scenarios, fire images at different scales and distances in both outdoor and indoor scenarios are selected for detection, and the detection results are compared with the SSD, Faster-RCNN and YOLOv5 algorithms.

(1) *Comparison of Different Methods in Outdoor and Indoor Scenarios*

**Figure 8:**
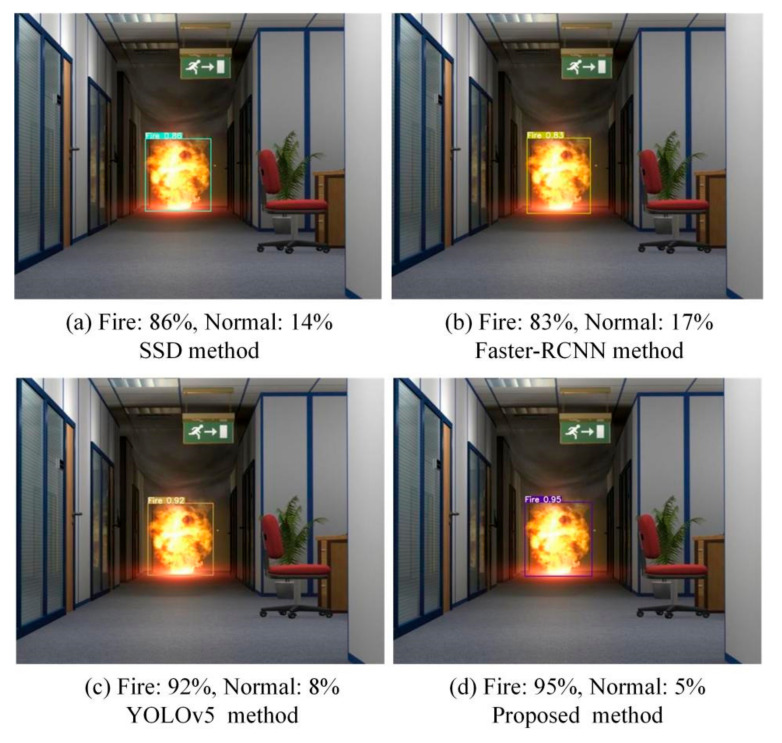
Indoor fire detection.

Figure 8 shows the fire detection results of the four methods in a bright indoor environment. The detection accuracy of the SSD and Faster-RCNN algorithms are 86% and 83%, respectively. Compared with the SSD and Faster-RCNN algorithms, the detection accuracy of the YOLOv5 algorithm is greatly improved and achieves 92%. Compared with the YOLOv5 algorithm, the detection accuracy of the proposed method improves by 3% and achieves 95% detection accuracy.

**Figure 9:**
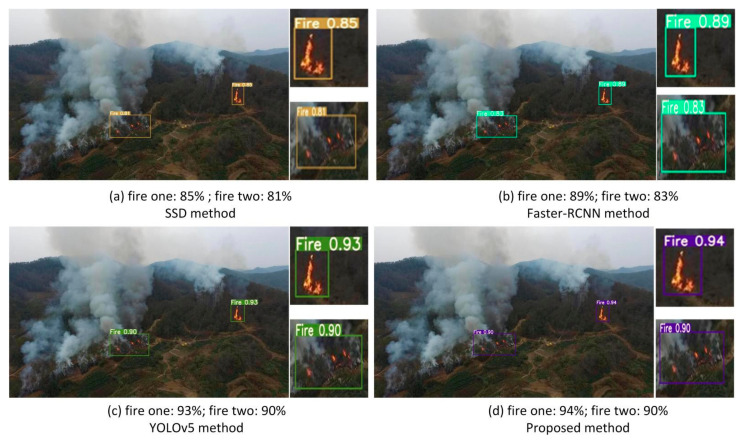
Outdoor fire detection.

Figure 9 shows the fire detection results for the four methods in an outdoor forest environment. It is clearly visible that there are two main areas of fire on the mountain, one higher set of flames in the upper right of the image and another lower set of flames in the lower middle of the image. All four methods can detect the two main areas of fire. However, the accuracy of these four methods and the size of the detected areas are not the same. The SSD algorithm detects two main regions, and its detection accuracies are 85% and 81%, respectively. The bounding box of the SSD algorithm’s detection results successfully covers the area of the higher flame but only covers a very small area of the lower flame and misses part of the area of the lower flame. The detection results of Faster-RCNN are similar to that of the SSD algorithm. For the higher flame detection, the detection accuracy of Faster-RCNN is slightly higher than that of the SSD algorithm at 89%. Although the detection accuracy of these two methods is almost the same for the lower flame, the detection coverage area of Faster-RCNN is larger than that of SSD. The detection accuracy of YOLOv5 is significantly superior to that of Faster-RCNN and SSD. Additionally, the bounding box of YOLOv5 and of the proposed method covers almost the entire area of the lower flame. This illustrates that the two superior methods, YOLOv5 and the proposed method, both successfully detect almost all on the mountain. However, the detection accuracy of the proposed method is 94% and superior to that of the YOLOv5 method.

(2) *Comparison of Different Methods for Different Distances*

**Figure 10:**
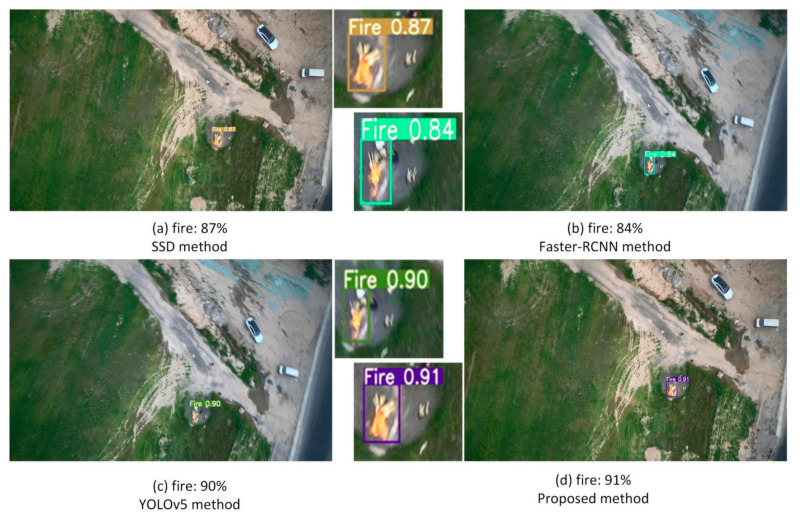
Fire detection at a long distance.

For fire detection at a long distance, a camera carried by a UAV is used to detect fires on the ground, as shown in Figure 10. From the detection results in Figure 10, it can be seen that the bounding boxes of the four methods successfully cover the fire on the ground. The detection accuracies of SSD and Faster-RCNN are lower at 87% and 84%, respectively. The Faster-RCNN method has the lowest detection accuracy mainly because the feature map extracted by Faster-RCNN is single-layer and lower resolution. The detection accuracy of the YOLOv5 method is better than SSD and Faster-RCNN and achieves 90%. The detection accuracy of the proposed method is superior to the YOLOv5 and achieves 91% detection accuracy. The accuracy of the proposed method is still above 90%, even when the fire target in the image is very small. The reason for the high detection accuracy of the proposed method is that the various scale features can be extracted by the proposed method according to the size of the object. The process of extracting fine-grained features by dynamic convolution YOLOV5 is enhanced; as a result, the detection accuracy is improved.

**Figure 11:**
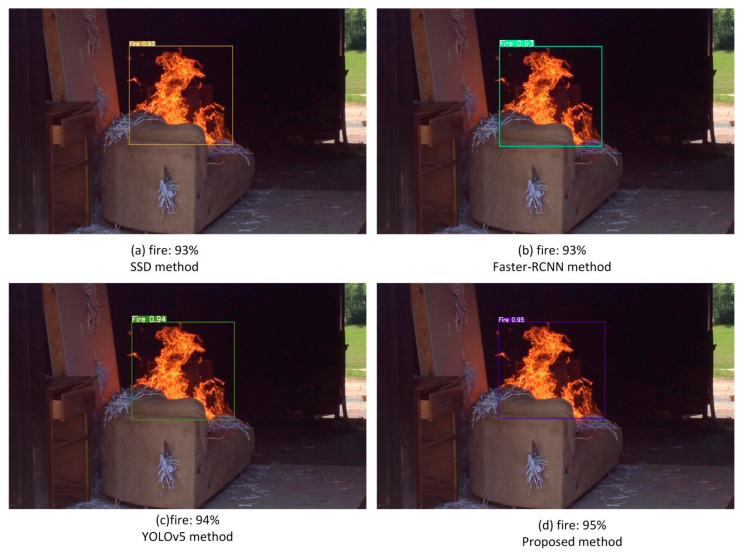
Fire detection at a short distance.

Figure 11 shows fire detection at a short distance; all four methods can accurately detect the fire on the sofa. The detection accuracies of SSD and Faster-RCNN are more than 90%; the main reason is that the fire object in the image is larger due to the short distance. The detection accuracy of YOLOv5 is much higher than SSD and Faster-RCNN and is similar to that of the proposed method; both are greater than 93%.

(3) *Comparison of Different Methods in the Field of Multi-Objective Detection*

**Figure 12:**
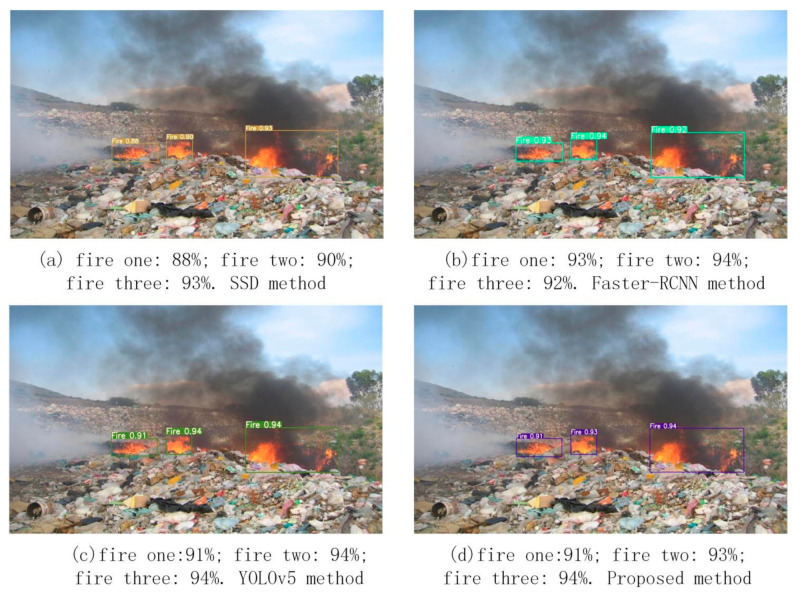
Multi-objective fire detection.

Figure 12 shows multiple fires at a garbage dump and the fire detection results for the four methods. It is clear that there are three main fires at the garbage dump. Each of the four methods generates the three bounding boxes, and they all cover the three main fires. However, the detection accuracies of Faster-RCNN, YOLOv5 and the proposed method are greater than 90% in all three fire regions. The detection accuracy of the SSD method is lower than 90% in the first fire region, mainly because the SSD model uses a multi-scale feature map to predict objects. High-level feature information with a large receptive field is used to predict large objects, and low-level feature information with a small receptive field is used to predict small objects. For the detection of multiple fires, the detection accuracy of Faster-RCNN is almost the same as YOLOv5 and the proposed method. Their detection accuracies are all greater than 90%. However, the proposed method’s detection accuracy for the three main fires is more stable than that of the Faster-RCNN and YOLOv5 algorithms. In Figure 12, it is difficult to distinguish which of the four methods has the best detection result. The major reason is that the fires in the three regions are clear and seen at a short distance.

(4) *Comparison of Different Methods in Different Weather Conditions*

**Figure 13:**
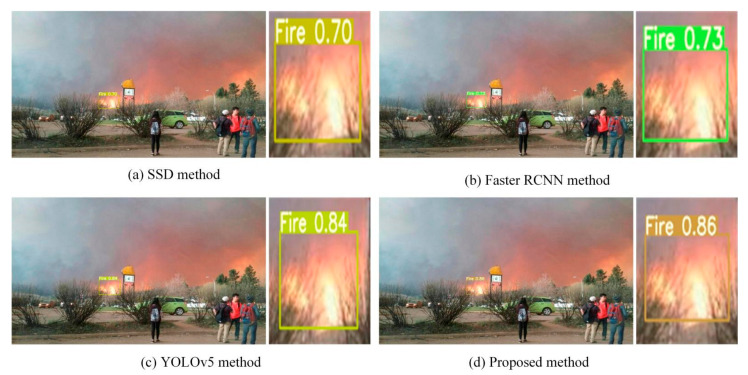
Fire detection of different methods on rainy days.

On a rainy day, we use the SSD, Faster-RCNN, YOLOv5 and proposed methods to detect fire, as shown in Figure 13. The fire detection accuracy of SSD is similar to that of the Faster-RCNN method; both are lower than 80%. According to the above analysis, it can be seen that the accuracy of fire detection using these two methods is generally higher than 80%. The major reason for the worse result in this case is that the rainy day influences the camera’s resolution and thus affects the ability of these two methods to extract flame features. As a result, these two methods cannot obtain high fire detection accuracy. However, the detection accuracies of both YOLOv5 and the proposed method are greater than 80%. Although the light rain influences the brightness and clarity of the image, the effectiveness of fire detection is not affected. The reason for this phenomenon is that the YOLOv5 and proposed methods both have a better ability to extract fine-grained targets from fire. Therefore, the fire detection performance of these two methods is not affected by the weather. 

#### 4.4.3. Comparison of Different Methods Based on Quantitative Evaluation

In order to fully verify the performance of the proposed method, we compare the proposed method with SSD, Fast R-CNN, Faster R-CNN, Cascade R-CNN and YOLOv5. Table 5 shows the detection results of the six methods using the 8280 images.

Fast-RCNN has the lowest TP and TN: almost 1000 fire and non-fire images cannot be detected, indicating that the method fails to detect many positive and negative samples. Additionally, the FP and FN of Fast-RCN are the highest among the six methods. This indicates that the Fast-RCNN method detects a large number of non-fire images as fire images and a large number of fire images as non-fire images. From its detection outcomes (TP, TN, FP and FN), the detection effectiveness of SSD is similar to that of Fast-RCNN. The SSD method also results in a large number of error detections. From Table 5, it is clearly visible that the detection capabilities of the Fast-RCNN and SSD methods are inferior to the other four methods. The TP of Faster-RCNN is similar to Cascade R-CNN and YOLOv5. However, the FN of Faster-RCNN is higher than those of Cascade R-CNN and YOLOv5. This illustrates that Faster-RCNN mistakes more fire images for non-fire images than Cascade R-CNN and YOLOv5. The TP and FN of Cascade R-CNN are almost the same as those of YOLOv5. However, the TN and FP of Cascade R-CNN are lower than those of YOLOv5. It shows that the fire identification performance of Cascade R-CNN is similar to that of YOLOv5. From Table 5, it is clearly visible that the performance of YOLOv5 is similar to that of the proposed method and superior to that of the SSD, Fast R-CNN, Faster R-CNN and Cascade R-CNN methods. In particular, the FN of YOLOv5 is much lower than those of Fast-RCNN and SSD. This indicates that a large number of fires can be detected by YOLOv5. The FN of the proposed method is the lowest, and its TP is the highest. This indicates that only a few fires cannot be detected; almost all fires can be detected by the proposed method. The dynamic convolution of the proposed method takes into account the dynamic characteristic of fire. As a consequence, both the TP and TN values for the proposed method are high. This indicates that the proposed method can accurately detect fire and that the detection performance of the proposed method is superior to that of other methods. 

According to the TP, TN, FP and FN, the four evaluation indexes are calculated, as shown in Table 6. It can be seen that the precision, recall, accuracy and F1-score of Fast-RCNN are the lowest among the four methods. The precision of SSD is similar to that of Fast-RCNN, mainly because precision only relates to TP and FP, as indicated by Equation (1). However, the recall of SSD is greater than that of Fast-RCNN because recall is related not only to TP but also to FN. As a result, its FN value leads to a higher detection accuracy for SSD than for Fast-RCNN. Although SSD’s accuracy of fire detection is better than Fast-RCNN’s, it is inferior to that of Faster-RCNN. The precision and recall of Faster-RCNN are slightly lower than those of Cascade R-CNN. Similarly, the accuracy and F1-score of Cascade R-CNN are slightly higher than those of Faster-RCNN. The major reason is that the TP, FP, TN and FN obtained by these two methods are basically the same. As a result, the fire identification performance of the two methods is almost the same. By the same token, the precision, recall, accuracy and F1-score of Cascade R-CNN are slightly lower than those of YOLOv5. This illustrates that the performance of Cascade R-CNN is slightly inferior to that of YOLOv5. This is because the number of low-level feature convolution layers is fewer when the Cascade R-CNN model uses the low-level feature information of the small receptive field to predict a small object. Compared with Fast-RCNN and SSD, the precision, recall, accuracy and F1-score of YOLOv5 are all higher than the scores for those two methods. The reason for this result is that YOLOv5 uses mosaic data augmentation. The proposed method uses dynamic convolution to replace the traditional static convolution kernel and extracts various scale features according to the size of objects. Therefore, the performance of object feature extraction and the accuracy of object detection are improved. Ultimately, the precision, recall, accuracy and F1-score of the proposed method are superior to YOLOv5. Compared to YOLOv5, the precision, recall, accuracy and F1-score of the proposed method are increased by 5.5%, 1.6%, 4.8% and 1.8%, respectively. In a word, the fire detection effectiveness of the proposed method is better than the other five deep learning methods. The major reason is that the proposed method replaces static convolution with dynamic convolution, which can extract features of every scale according to the object size. The reason for doing this is to increase the fine-grained feature extraction ability, reducing the number of false positives. Additionally, dilated convolution can increase the receptive field of the network without changing the network resolution and then capture multi-scale context information. Thus, the detection accuracy for multiple and small objects is improved without affecting the detection speed. 

**Figure 14:**
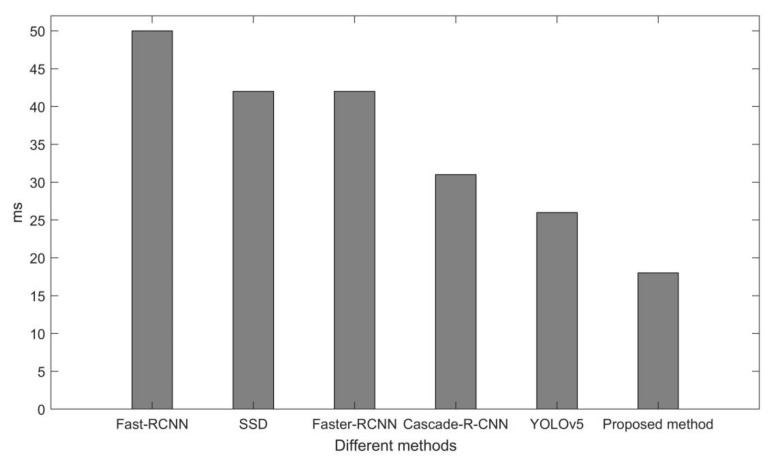
Processing time of each image by different methods.

The processing time of each image in the video stream by different methods is obtained, as shown in Figure 14.

Figure 14 shows that the Fast-RCNN method requires the longest processing time for each image, and the proposed method requires the shortest processing time. The processing time of each image for the proposed method and the YOLOv5 method is much lower than for the other four methods. Fast-RCNN takes almost 50 ms to process each image. SSD and Faster-RCNN method both take about 40 ms to process each image. Cascade R-CNN takes about 30 ms to process each image. The proposed method takes only 18 ms to process each image and thus requires the least amount of time. 

## 5. Conclusions

With the gradual development of intelligent monitoring systems, it is important to detect the initial flames of a fire and then control its spread and avoid casualties. Based on the advanced YOLOv5 model used in the field of target detection, a dynamic convolution YOLOv5 model is applied to flame detection and verified on a self-made flame image dataset. First, the K-means++ algorithm is used to update anchor boxes. Second, dynamic convolution is introduced to replace the traditional static convolution kernel. The feature values of various scales are extracted according to the target sizes, which improves the ability of the network to extract target features and reduces the false detection rate. Finally, the network structure is pruned to reduce the calculation costs of the model and improve detection speed. Experiments show that the dynamic convolution YOLOv5 model proposed in this paper achieves good fire detection effectiveness and, in particular, significantly improves the detection of small targets. It not only ensures the accurate detection of fire but it also realizes real-time detection.

## Figures and Tables

**Table 1:** Experimental dataset.

Dataset	Fire Images	Non-Fire Images	Total
Train set	8054	6046	14,100
Validation set	2033	1521	3554
Test set	5150	3130	8280
Total	15,237	10,697	25,934

**Table 2:** Four possible outcomes of fire identification.

	Negative	Positive
False	False Negative (FN)	False Positive (FP)
True	True Negative (TN)	True Positive (TP)

**Table 3:** Comparison results of the dynamic convolution experiment.

	P	R	Acc	F1-Score	Detection Time (ms)
YOLOv5	89.7%	97.4%	91.5%	46.7%	26
Dynamic convolution YOLOv5	96.4%	99%	96.8%	49.3%	29

**Table 4:** Comparison results before and after pruning.

	P	R	Acc	F1-Score	Model Size	Detection Time (ms)
YOLOv5	89.7%	97.4%	91.5%	46.7%	13.7	26
YOLOv5 (after pruning)	88.2%	96.6%	89.7%	44.3%	10.8	13

**Table 5:** Detection results of different algorithms.

Different Methods	TP	TN	FP	FN
Fast-RCNN	4314	2290	840	836
SSD	4501	2391	739	649
Faster-RCNN	4972	2417	713	178
Cascade R-CNN	5003	2447	683	147
YOLOv5	5020	2554	576	130
Proposed Method	5100	2873	257	50

**Table 6:** Quadri-partite measures of different methods.

Different Methods	P (%)	R (%)	Acc (%)	F1-Score (%)
Fast-RCNN	83.7	83.8	79.8	41.9
SSD	85.9	87.3	83.2	43.3
Faster-RCNN	87.5	96.5	89.2	44.8
Cascade R-CNN	88.1	97.1	90.1	45.6
YOLOv5	89.7	97.4	91.5	46.7
Proposed method	95.2	99	96.3	48.5

## Data Availability

The data used to support the findings of this study are available from the corresponding author upon request.
